# MiR-613 inhibits the proliferation, migration, and invasion of papillary thyroid carcinoma cells by directly targeting TAGLN2

**DOI:** 10.1186/s12935-021-02083-8

**Published:** 2021-09-16

**Authors:** Yonglian Huang, Hengwei Zhang, Lidong Wang, Chenxi Liu, Mingyue Guo, Hao Tan, Zhen Liu

**Affiliations:** 1 Department of General Surgery, Shengjing Hospital of China Medical University, No. 36 Sanhao Street, Shenyang, 110004 China; 2 Department of General Surgery, The First Hospital of Lanzhou University, 1 Donggang West Rd, Lanzhou, 730000 China

**Keywords:** Thyroid neoplasm, miR-613, TAGLN2, EMT

## Abstract

**Background:**

Papillary thyroid carcinoma (PTC), with a rapidly increasing incidence, is the most prevalent malignant cancer of the thyroid. However, its pathogenesis is unclear and its specific clinical indicators have not yet been identified. There is increasing evidence that microRNAs (miRNAs) play important roles in tumor occurrence and progression. Specifically, miR-613 participates in the regulation of tumor development in various cancers; however, its effects and mechanisms of action in PTC are still unclear. Therefore, in this study, we investigated the expression and function of miR-613 in PTC.

**Methods:**

qRT-PCR was used to determine miR-613 expression in 107 pairs of PTC and adjacent-normal tissues as well as in PTC cell lines and to detect *TAGLN2* mRNA expression in PTC tissues and adjacent normal tissues. Western blot analysis was performed to identify TAGLN2 and epithelial–mesenchymal transition (EMT) biomarkers. The effects of miR-613 on PTC progression were evaluated by performing MTS, wound-healing, and Transwell assays in vitro. Luciferase reporter assays were also performed to validate the target of miR-613.

**Results:**

In PTC, miR-613 was significantly downregulated and its low expression level was associated with cervical lymph node metastasis. However, its overexpression significantly suppressed PTC cell proliferation, migration, and invasion and inhibited EMT. *TAGLN2* was identified as a target of miR-613, which also significantly inhibited the expression of TAGLN2. Further, the restoration of TAGLN2 expression attenuated the inhibitory effects of miR-613 on PTC cell proliferation and metastasis.

**Conclusion:**

Our findings demonstrated that miR-613 can suppress the progression of PTC cells by targeting *TAGLN2*, indicating that miR-613 plays the role of a tumor suppressor in PTC. Overall, these results suggest that the upregulation of miR-613 is a promising therapeutic strategy for PTC.

**Supplementary Information:**

The online version contains supplementary material available at 10.1186/s12935-021-02083-8.

## Background

Papillary thyroid cancer (PTC) is the most frequently encountered type of thyroid malignancy, and over the past few decades, there has been a remarkable increase in its incidence [1, 2]. Primarily, PTC is treated via thyroidectomy (with or without neck dissection) or via adjuvant radioactive iodine [3] and thyroid stimulating hormone suppression therapies [4]. Although PTC has a favorable prognosis, certain cases show cervical lymph node metastasis at an early stage as well as a significant risk of recurrence [5], which is the main cause of death in patients with PTC. Moreover, given that no specific molecular biomarkers for PTC have been identified, monitoring its progression and prognosis is still challenging. Several risk factors, including obesity [6], radiation exposure [7], iodine deficiency or excess [8], and genetic factors [9] are associated with PTC. Therefore, clarifying the molecular mechanisms underlying its tumorigenesis and progression are necessary in order to establish a reliable basis for clinical diagnosis and treatment.

MicroRNAs (miRNAs) are a class of highly conserved small non-coding RNAs that negatively regulate gene expression by completely or incompletely binding to the 3′ untranslated region (3′-UTR) of target mRNAs [10]. Rarely, miRNAs may bind to the 5′ UTR segment of target mRNAs and enhance their translation [11]. Reportedly, their dysregulation is involved in several biological and pathological processes, such as cell differentiation [12], proliferation, migration [13], apoptosis, oxidative damage [14], inflammation [15], immune escape [16], and tumorigenesis. Emerging evidence has also indicated that alterations in miRNA expression levels play critical roles in the occurrence and development of various cancers, including ovarian cancer [17], triple-negative breast cancer [18], gastric cancer [19], and colon cancer [20]. Additionally, miRNAs are considered to be potential biomarkers for the early diagnosis and prognostic evaluation of cancer. Among the numerous miRNAs that exist, the roles of miR-613 in the regulation of tumor development are well-established. First, its involvement in cell metabolism by targeting liver X receptor (LXR) gene has been reported [21]. Second, its downregulation in osteosarcoma [22], glioma [23], and non-small-cell lung cancer [24], and upregulation in cervical cancer [25] and colorectal cancer [26] have also been demonstrated. Further, it has also been demonstrated that miR-613, which is downregulated in laryngeal squamous carcinoma, can function as a tumor-suppressor gene by downregulating the expression of PDK1 [27]. Furthermore, its expression decreases in triple-negative breast cancer and restoration to initial levels can repress the migration and invasion of breast cancer cells via the inhibition of Daam 1 expression [28]. It has also been demonstrated that miR-613 enhances the sensitivity of gastric [29] and liver cancer cells [30] to chemotherapy, and its expression is significantly decreased in radiotherapy-resistant non-small-cell lung cancer [31]. Therefore, based on these previous studies, miR-613 plays an important role in regulating tumor cell malignancy. However, the mechanism by which it influences tumorigenesis in PTC is still unclear.

TAGLN2, also known as transgelin-2, which contains an N-terminal calponin homolog (CH) domain and a C-terminal calponin-like domain, belongs to the family of actin-binding proteins. Specifically, it binds to actin to facilitate the formation of cytoskeletal structures, a process that plays a fundamental role in various biological processes. Furthermore, TAGLN2 also participates in the formation of an immunological synapse in T cells, maintains F-actin content, and blocks actin depolymerization, in a process similar to the formation of aggressive pseudopodia in malignant tumors [32–34]. It has also been observed that TAGLN2 is dysregulated in several malignancies and is involved in cancer cell proliferation, invasiveness, apoptosis, and metastasis [35–37]. For example, the upregulation of TAGLN2 triggers the proliferation, migration, and invasion of esophageal squamous cell cancer cells [38]. Furthermore, TAGLN2 is overexpressed in lung cancer tissues, and its high expression is closely related to the clinical stages and lymph node metastasis of lung cancer [39]. However, its role in the tumorigenesis and progression of PTC is still unknown.

Given that miRNAs are posttranscriptional regulators of gene expression, their dysregulation contributes to the progression of PTC via the regulation of downstream target molecules. For example, the restoration of miR-214 expression in PTC can inhibit cell proliferation, migration, and invasion and also induce apoptosis in vitro by targeting proteasome 26S subunit non-ATPase 10 (PSMD10) [40]. Additionally, the upregulation of miR-215 in PTC cells shows similar effects and remarkably represses metastasis by modulating epithelial–mesenchymal transition (EMT) via the ARFGEF1/AKT/GSK-3β signaling pathway [41]. However, the underlying mechanism and downstream targets of miR-613 in PTC are still unclear.

We speculated that the miR-613/TAGLN2 axis acts as a tumor suppressor in PTC. To verify this hypothesis, biological experiments were performed on cells grown in vitro. Thus, an inverse correlation was observed between miR-613 and TAGLN2, and the results provided evidence that miR-613 could inhibit PTC cell proliferation, migration, and invasion in vitro. Our results also clarify the cross-talk between miR-613 and TAGLN2, which could serve as a basis for the development of novel targeted therapies that involve miR-613.

## Methods

### Cell culture

Human thyroid follicular epithelial cell line Nthy-ori-3-1 (N3) and PTC cell line K1 were purchased from the European Collection of Authenticated Cell Cultures (ECACC). PTC cell lines TPC-1 and BCPAP were purchased from BeNa Culture Collection (BNCC) and authenticated via STR Genotyping. The K1 cells were cultured in a medium containing 10% fetal bovine serum (FBS; LONSERA, Ciudad de la Costa, Uruguay), 1% penicillin, and streptomycin (Gibco, Gaithersburg, MD, USA) mixed with MCD105 (Abcam, Cambridge, MA, USA), F-12, and DMEM (Hyclone, Logan, UT, USA). The Nthy-ori-3-1 cells were cultured in RPMI-1640 (Hyclone, Logan, UT, USA), while the TPC-1 and BCPAP cells were cultured in DMEM (Hyclone, Logan, UT, USA) supplemented with 10% FBS, 1% penicillin, and streptomycin. All the cells were cultured at 37 °C in a 5% CO_2_ atmosphere.

### Human samples

PTC tissues and adjacent normal tissues (confirmed via pathological diagnosis) were obtained from 107 patients at the Department of General Surgery, Shengjing Hospital of China Medical University. The patients did not receive any local or systemic treatments before the operation, and the adjacent normal tissues were collected at > 2 cm from the tumor edge. Immediately after surgical resection, all the collected tissues were stored in liquid nitrogen. Informed consent was obtained from patients or guardians, and the study was approved by the ethics committee of the hospital.

### Quantitative reverse-transcription polymerase chain reaction (qRT-PCR)

Total RNA extraction from cells and tissues was performed using the miRNeasy Mini Kit (Qiagen, Germantown, MD, USA), and non-coding small RNA reverse transcription was performed using the Hairpin-it™ microRNA and U6 snRNA Normalization RT-PCR Quantitation Kit (GenePharma, Shanghai, China). Complementary cDNA was obtained using the PrimeScript RT Reagent Kit (Takara, Dalian, China). Further, qRT-PCR analyses for the detection of miR-613 and *TAGLN2* were conducted using SYBR Premix Real-time PCR Reagent (Takara) and the ABI 7500 FAST Real Time PCR System (Applied Biosystems, Foster City, CA, USA). All the primers were purchased from GenePharma. The primer sequences were as follows: miR-613, forward primer 5′-CGGCCGCGAGGAATGTTCCTTC-3′, reverse primer 5′-ATCCAGTGCAGGGTCCGAGG-3′; and *TAGLN2*, forward primer 5′-ATCACCACCCAGTGCCGAAAG-3′, reverse primer 5′-CATGGTGGAGGCCTGGATCTT-3′. Further, the fold-change in the expression of each gene was determined using the 2^−ΔΔCt^ method. *U6* and *GAPDH* were chosen as reference genes for miR-613 and *TAGLN2*, respectively.

### Western blot analysis

Total proteins were extracted using RIPA buffer (with phenylmethylsulfonyl fluoride at 100:1). After the proteins were separated via sodium dodecyl sulfate–polyacrylamide gel electrophoresis, they were transferred onto polyvinylidene difluoride membranes. Subsequently, the membranes were incubated with 5% bovine serum albumin (Solarbio, Beijing, China) for 2 h at room temperature (22–25 ℃), followed by incubation with rabbit anti-TAGLN2 antibody (1:1000), rabbit anti-E-cadherin (1:2000), rabbit anti-N-cadherin (1:2000), rabbit anti-vimentin (1:2000), rabbit anti-MMP2 (1:800), and rabbit anti-GAPDH (1:8000) (all purchased from ProteinTech Group Inc., Wuhan, China) at 4 °C for 14–16 h. Thereafter, the collected membranes were washed with TBST (Tris-buffered saline, 0.1% Tween 20) three times. Horseradish peroxidase-labeled goat anti-rabbit antibody (1:2500; ProteinTech Group Inc.) or horseradish peroxidase-labeled goat anti-mouse antibody (1:10,000; Proteintech Group Inc.) was used as a secondary antibody, and after incubation at room temperature (22–25 ℃) for 2 h, the membranes were washed with TBST three times and detected via enhanced chemiluminescence.

### Cell transfection

Three PTC cell lines (K1, TPC-1, and BCPAP) were used for cell transfection. One day before transfection, the cells were seeded into 6-well plates. When the cell density reached 60–70% confluence, the cells were transfected with miR-613 mimics, miR-NC (negative control), pCMV-TAGLN2, and pCMV-control plasmids using Lipofectamine 3000 (Invitrogen, Carlsbad, CA, USA) according to the manufacturer’s instructions. The miR-613 mimics and miR-NC were purchased from GenePharma. Specifically, the sequence of the miR-613 mimics was 5′-AGGAAUGUUCCUUCUUUGCC-3′, and the final concentration was 100 nM. The pCMV-TAGLN2 and pCMV-control plasmids were purchased from GeneChem (Shanghai, China). The final transfection amount was 2500 ng. Further, qRT-PCR was performed to validate the efficiency of the transfection process.

### Dual-luciferase reporter assay

Four online databases, namely, TargetScan (http://www.targetscan.org/), miRDB (http://mirdb.org/), RNAInter (http://www.rna-society.org/rnainter/), and miRWalk (http://www.ma.uni-heidelberg.de/apps/zmf/mirwalk/), were used to predict the target genes of miR-613. The intersection of datasets was determined using a Venn Diagram, and the binding sites for TAGLN2 and miR-613 were determined using an online database, TargetScan. Specifically, after synthesis, human *TAGLN2* 3′-UTR (containing the miR-613 binding sequence, 5′-ACATCC-3′) and a mutant 3′-UTR (containing the mutant miR-613 binding site, 5′-TGTAAG-3′) were inserted into pmiR-RB-Report Vector (Ribo Bio Co., Guangzhou, China) for analysis.

Further, 293T cells were seeded into 96-well-plates at a density of 3000 cells per well on the day before transfection. When the cells reached 70–80% confluence, they were co-transfected with each luciferase plasmid and miR-613 mimics or miR-NC. Thereafter, a Luciferase Kit (Promega, Madison, WI, USA) was used to measure the fluorescence intensity after 48 h of transfection.

### MTS assay

PTC cell proliferation was evaluated using the CellTiter 96® AQueous One Solution Cell Proliferation Assay Kit (Promega, USA) according to the manufacturer’s protocol. Specifically, the cells transfected with miR-613 mimics or miR-NC were seeded in 96-well plates after 24 h and cultured for 24, 48, 72, and 96 h. Thereafter, 20 µL of MTS solution was added to each well, followed by incubation for 4 h at 37 °C. Absorbance was then detected at 490 nm using a multi-mode microplate reader. The experiments were performed at least in triplicates.

### Wound healing assay

Cell migration ability was determined via the wound healing assay. Specifically, transfected cells were cultured in 6-well plates until the cell density in the entire medium was close to 90%. Thereafter, the cell monolayer was scraped using a 200 µL pipette, and the collected cells were washed three times with phosphate buffered saline. This was followed by culturing in a medium containing 2% FBS for 24 h. Further, images corresponding to each scratch were obtained at 0 and 24 h in the same visual fields under an inverted microscope. Cell migration was then calculated as the percentage of wound closure as follows: wound closure (%) = [(scratch area at 0 h—scratch area at 24 h)/scratch area at 0 h] × 100. The experiments were repeated at least three times.

### Transwell assay

Transwell chambers (8 μm; Costar, Corning, New York City, NY, USA) containing Matrigel were employed to examine cell invasion ability. Briefly, 5 × 10^4^ cells in 200 µL of serum-free medium were transferred to the upper chambers and 600 µL of the medium with 10% FBS was added to the lower chambers. After 24–48 h of incubation at 37 °C in 5% CO_2_, the cells in the upper chambers were scraped, and invaded cells were fixed with 4% paraformaldehyde and stained with crystal violet. Thereafter, to count the cells that migrated through the membrane, five fields were randomly selected and observed under a microscope. The experiments were also repeated at least three times.

### Statistical analysis

The data corresponding to the clinical samples were presented as mean ± SEM, while other results were expressed as mean ± SD of triplicate experiments. Statistical analyses and the generation of graphs were performed using GraphPad 7.0. For comparisons, Student’s *t*-tests were performed and to evaluate the relationship between *miR-613* and *TAGLN2* mRNA expression in PTC tissues, Spearman’s correlation coefficients were determined. *p* < 0.05 was considered statistically significant.

## Results

### MiR-613 was downregulated in PTC tissues and cell lines

**Fig. 1:**
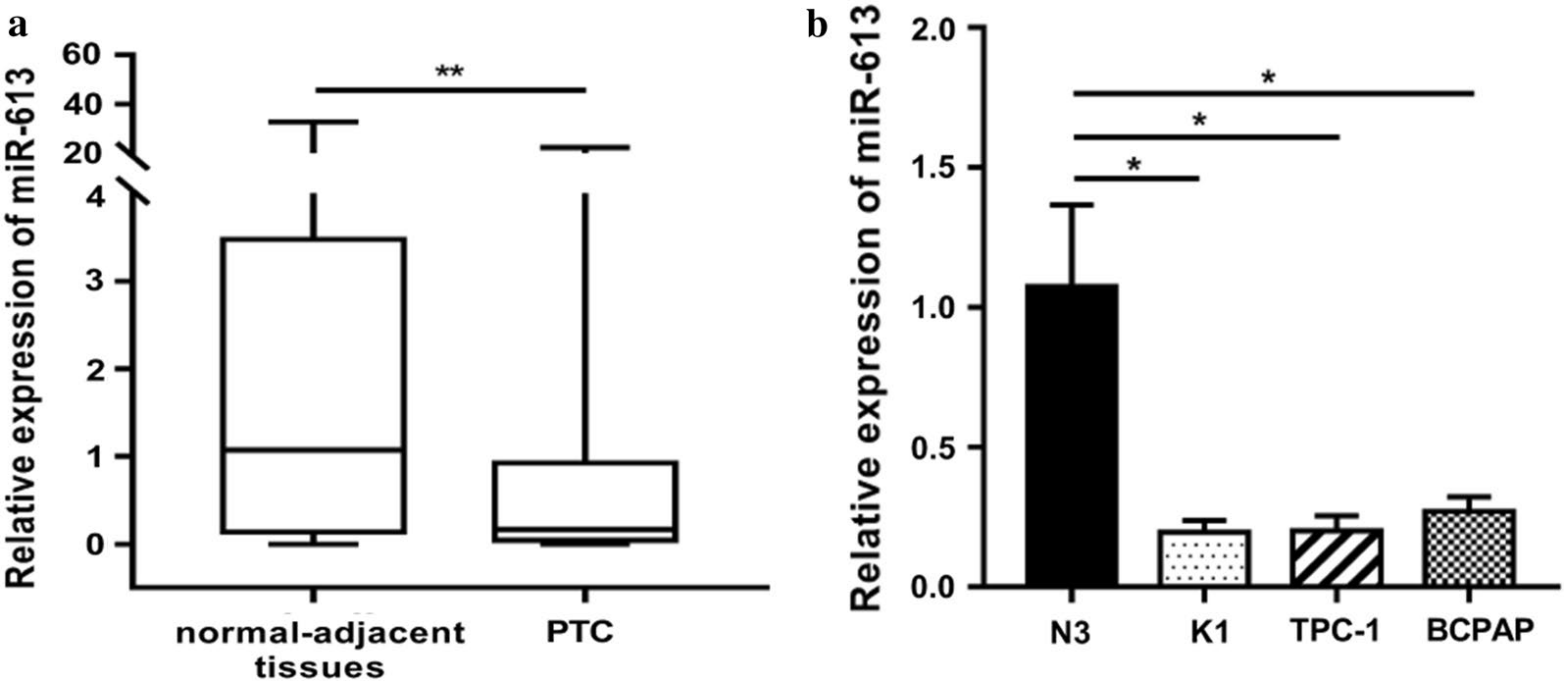
Abnormal downregulation of miR-613 in papillary thyroid cancer (PTC) tissues and cell lines. **a** Downregulation of miR-613 in PTC tissues compared with adjacent non-tumor tissues (n = 107) based on qRT-PCR analysis. **b** Downregulation of miR-613 in PTC cell lines (K1, TPC-1, and BCPAP cell lines) compared with the thyroid follicular epithelial cell line (N3)**.** * p < 0.05, ** *p* < 0.01, *** p < 0.001, **** p < 0.0001

The expression of miR-613 was evaluated in 107 pairs of PTC and normal-adjacent tissues, and it was observed that the levels of miR-613 in PTC tissues (1.339 ± 0.523) were lower than those in the normal-adjacent tissues (3.976 ± 0.934) (p < 0.05; Fig. 1a). Consistent with the clinical data, the levels of miR-613 expression were lower in all the PTC cell lines than in the Nthy-ori-3–1 cell lines (p < 0.05; Fig. 1b). Further, the level of miR-613 expression was closely related to lymph node metastasis in patients with PTC (Table 1). It was also observed that the relative expression level of miR-613 in patients without lymph node metastasis (1.689 ± 0.057) was significantly higher than that in patients with lymph node metastasis (0.326 ± 0.040). These results indicated that miR-613 possibly functioned as a tumor suppressor in PTC.

**Table 1:** Correlation between miR-613 expression level in PTC and clinicopathologic features

Clinicopathologic features	Cases	miR-613 expression	P value
Gender			
Male	86	1.471 ± 0.188	0.415
Female	21	1.304 ± 0.103	
Age			
≥ 55	83	1.358 ± 0.101	0.583
< 55	24	1.251 ± 0.207	
Tumor size			
1–2 cm	64	1.4 ± 0.145	0.656
> 2 cm	43	1.329 ± 0.040	
Lymph node metastasis			
Yes	79	0.326 ± 0.040	0.01
No	28	1.689 ± 0.057	
Multiplicity			
Yes	57	1.3 ± 0.127	0.513
No	50	1.424 ± 0.135	
Extra thyroid invasion			
Yes	32	1.496 ± 0.155	0.418
No	75	1.236 ± 0.143	

### Suppression of PTC cell proliferation, migration, and invasion by MiR-613

**Fig. 2:**
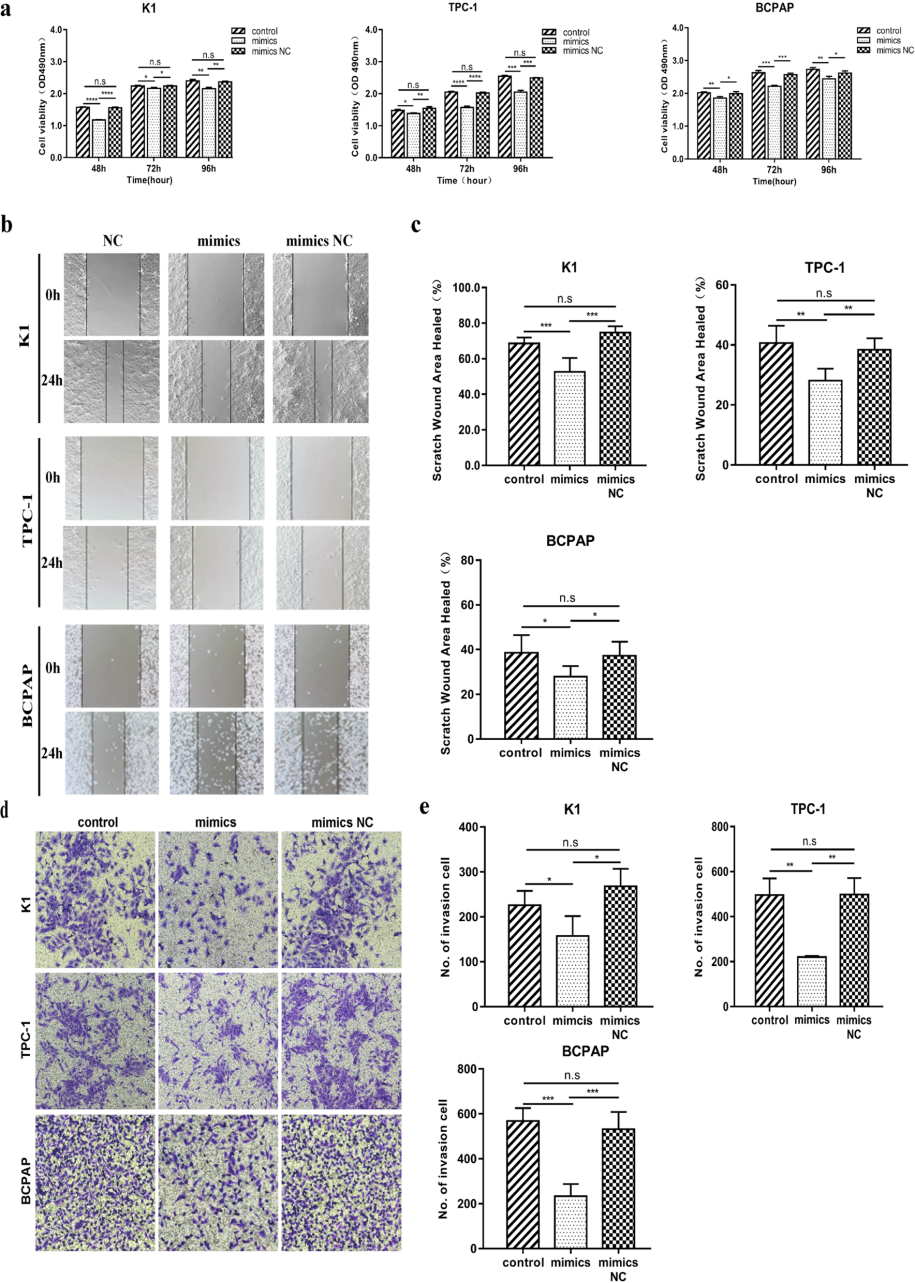
Inhibition of papillary thyroid cancer (PTC) cell proliferation, migration, and invasion by miR-613 in vitro. K1, TPC-1, and BCPAP cells were transfected with miR-613 mimics or miR-NC. **a** Significant inhibition of PTC cell growth at 48, 72, and 96 h owing to miR-613 restoration. **b**, **c** Inhibition of PTC cell migration ability via miR-613 restoration based on wound healing assays. Left, representative images showing the migration of three PTC cells in six-well culture plates (b). Right, quantification of the cell migration distances corresponding to three PTC cells (c). **d**, **e** Restoration of miR-613 inhibited PTC cell invasion. Images of cells that migrated to the bottom chamber (**d**) and calculated number of invasion cells (**e**). * p < 0.05, **p < 0.01, *** p < 0.001, **** p < 0.0001

Based on functional analyses, miR-613 was overexpressed in PTC cells owing to transfection with miR-613 mimics. Based on MTS assay, it was observed that the overexpression of miR-613 resulted in a significant decrease in PTC cell growth (Fig. 2a and Additional file 1: Fig. S1). Further, to confirm the effects of miR-613 on PTC cell migration and invasion, wound-healing and Transwell assays were performed. The wound-healing assay showed that miR-613 mimics inhibited PTC cell migration (Fig. 2b and c), and as shown in Fig. 2d and e, the Transwell assay indicated that the upregulation of miR-613 inhibited PTC cell invasion. Further, the results of migration and invasion assays suggested that miR-613 acts as an anti-oncogene by suppressing PTC cell migration and invasion (Table 2).

**Table 2:** The healing area and numbers of invasion cells in group of PTC cells transfected with mimics or mimics NC

Cell lines	Scratch wound area healed	P value	Number of invasion cells	P value
K1				
Control	68.6% ± 1.4%	0.01^a^	225.8 ± 14.3	0.043^a^
Mimics	52.6% ± 3.2%	0.000^b^	157.3 ± 25.7	0.032^b^
Mimics NC	74.7% ± 1.8%	0.063^c^	268.0 ± 22.5	0.145^c^
TPC-1				
Control	40.7% ± 2.3%	0.001^a^	496.2 ± 73.5	0.006^a^
Mimics	28.1% ± 1.6%	0.001^b^	220.2 ± 5.4	0.006^b^
Mimics NC	38.4% ± 1.5%	0.442^c^	498.2 ± 73.7	0.985^c^
BCPAP				
Control	38.6% ± 3.6%	0.023^a^	568.6 ± 25.6	0.000^a^
Mimics	28.0% ± 1.9%	0.021^b^	233.8 ± 24.2	0.000^b^
Mimics NC	37.2% ± 2.8%	0.776^c^	523.0 ± 34.1	0.416^c^

^a^Control vs. mimics

^b^Mimics vs. mimics NC

^c^Control vs. mimics NC

### Inhibition of EMT induction in PTC cells by MiR-613

**Fig. 3:**
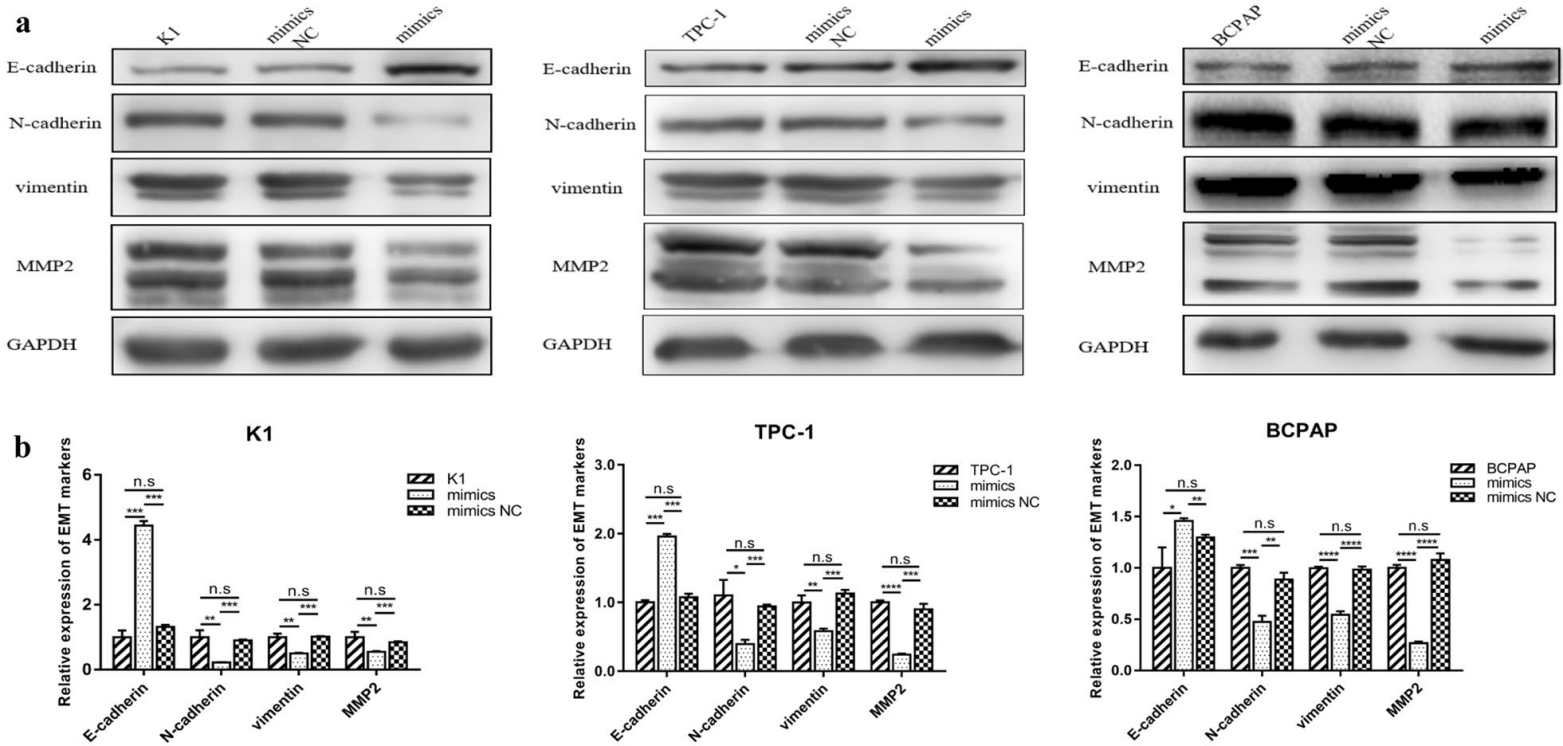
Inhibition of EMT induction by miR-613 in papillary thyroid cancer (PTC) cells. **A** Increased E-cadherin expression, and decreased N-cadherin, Vimentin, and MMP2 expression based on western blot analysis. **b** Relative expression levels of EMT-related markers. * p < 0.05, **p < 0.01, *** p < 0.001, **** p < 0.0001

It has been previously demonstrated that miRNAs suppress invasion and metastasis via EMT mediation. To determine whether miR-613 has inhibitory effects on EMT in PTC, we examined the expression of the proteins of the epithelial marker, E-cadherin, and the mesenchymal markers, N-cadherin, vimentin, and MMP2 in PTC cells. As shown in Fig. 3, the protein expression levels of E-cadherin were significantly higher in PTC cells with miR-613 upregulation than in the control groups, while the expression levels of N-cadherin, vimentin, and MMP2 were significantly lower. Thus, miR-613 inhibited PTC cell invasion and metastasis via the regulation of EMT in the PTC cells.

### *TAGLN2* as a direct target of miR-613

**Fig. 4:**
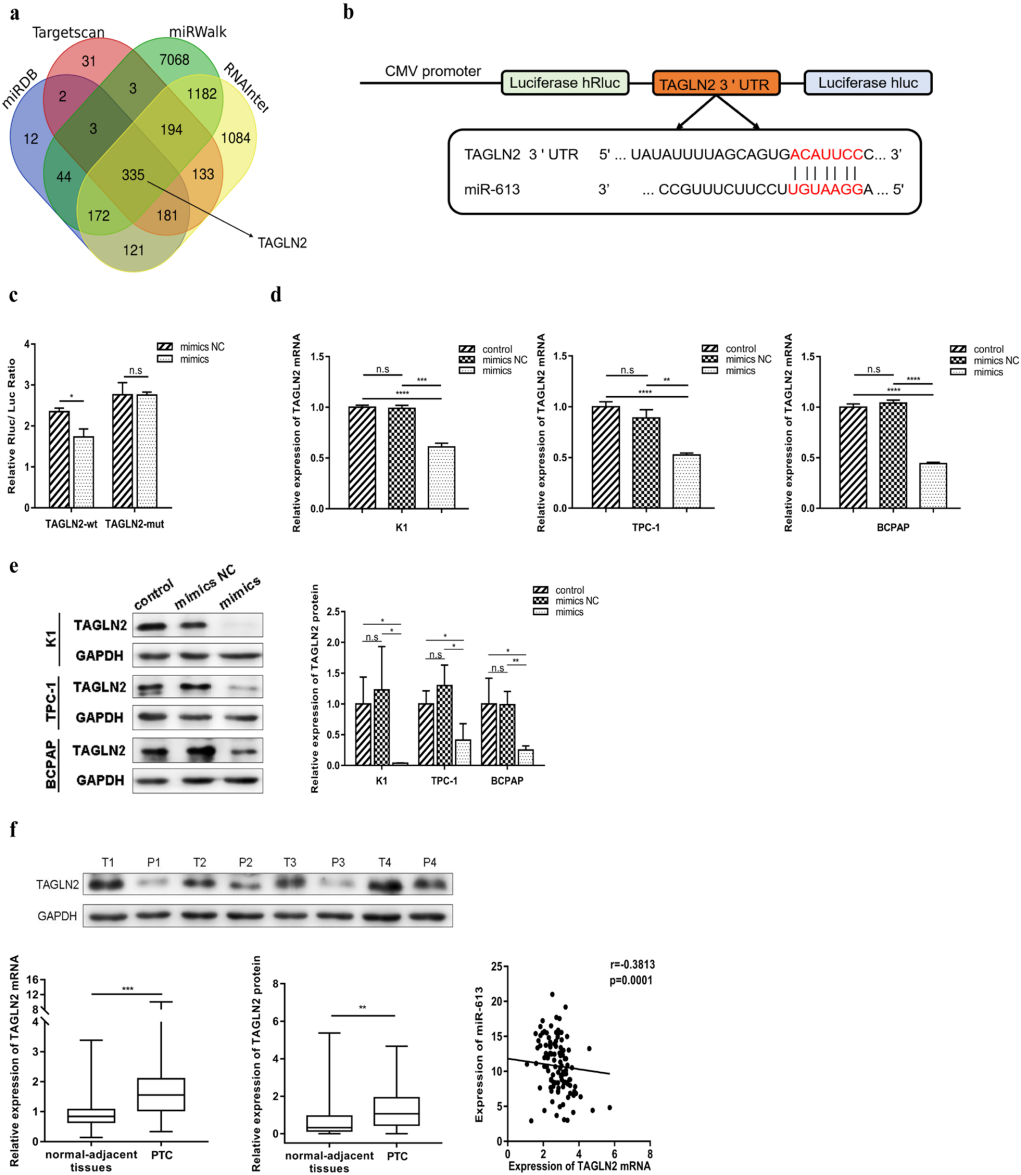
TAGLN2 as a direct target of miR-613. **a** Venn diagram displaying miR-613 predicated to target TAGLN2 based on four different predication algorithms, namely, TargetScan, miRDAB, RNAInter, and miRWalk. **b** Schematic representation of TAGLN2 showing the putative miRNA target site. **c** Suppression of the luciferase activity of TAGLN2-3`UTR by miR-613 in 293 T cells and removal of the miR-613-mediated inhibitory effect by TAGLN2-3`UTR-mut.** d** Decrease in TAGLN2 mRNA levels owing to the overexpression of miR-613 based on qRT-PCR analysis. **e** Decrease in TAGLN2 protein level owing to the overexpression of miR-613 based on western blot analysis. **f** Upregulation of TAGLN2 in papillary thyroid cancer (PTC) tissues compared with adjacent non-tumor tissues based on qRT-PCR and western blot analyses. The expression of miR-613 was inversely correlated with the expression of TAGLN2 mRNA. * p < 0.05, **p < 0.01, *** p < 0.001, **** p < 0.0001

To predict the downstream targets of miR-613, four online databases were used. Thus, it was observed that miR-613 directly targets *TAGLN2* by binding to its 3′‐UTR segment at positions 72–78 and 186–192. Further, to confirm that miR-613 targets the *TAGLN2* 3′‐UTR segment, a fragment including the miR-613 binding site within *TAGLN2*, was cloned into a vector (WT-TAGLN2) (Fig. 4a and b). A mutant recombinant, in which the seed regions of miRNA binding sites were mutated (MUT-TAGLN2), was also constructed. Thereafter, 293T cells were co-transfected with RNA oligos and pri-recombinants. In this regard, reporter assays showed that miR-613 overexpression inhibited the luciferase activity of WT-TAGLN2. However, no significant effect was observed when the cells were co-transfected with MUT-TAGLN2 (Fig. 4c). Additionally, qRT-PCR and western blot analysis showed that the overexpression of miR-613 significantly decreased the expression of TAGLN2 at both mRNA and protein levels in K1, TPC-1, and BCPAP cells (Fig. 4d and e). The further evaluation of TAGLN2 expression in PTC issues showed that its mRNA and protein levels were significantly higher in PTC tissues than in the normal-adjacent tissues (Fig. 4f). Further, Spearman’s correlation analysis revealed a significant inverse correlation between *TAGLN2* and miR-613 (Fig. 4f). Thus, we confirmed that *TAGLN2* is a direct target of miR-613.

### Offset of the inhibitory effect of miR-613 in PTC cells by TAGLN2

**Fig. 5:**
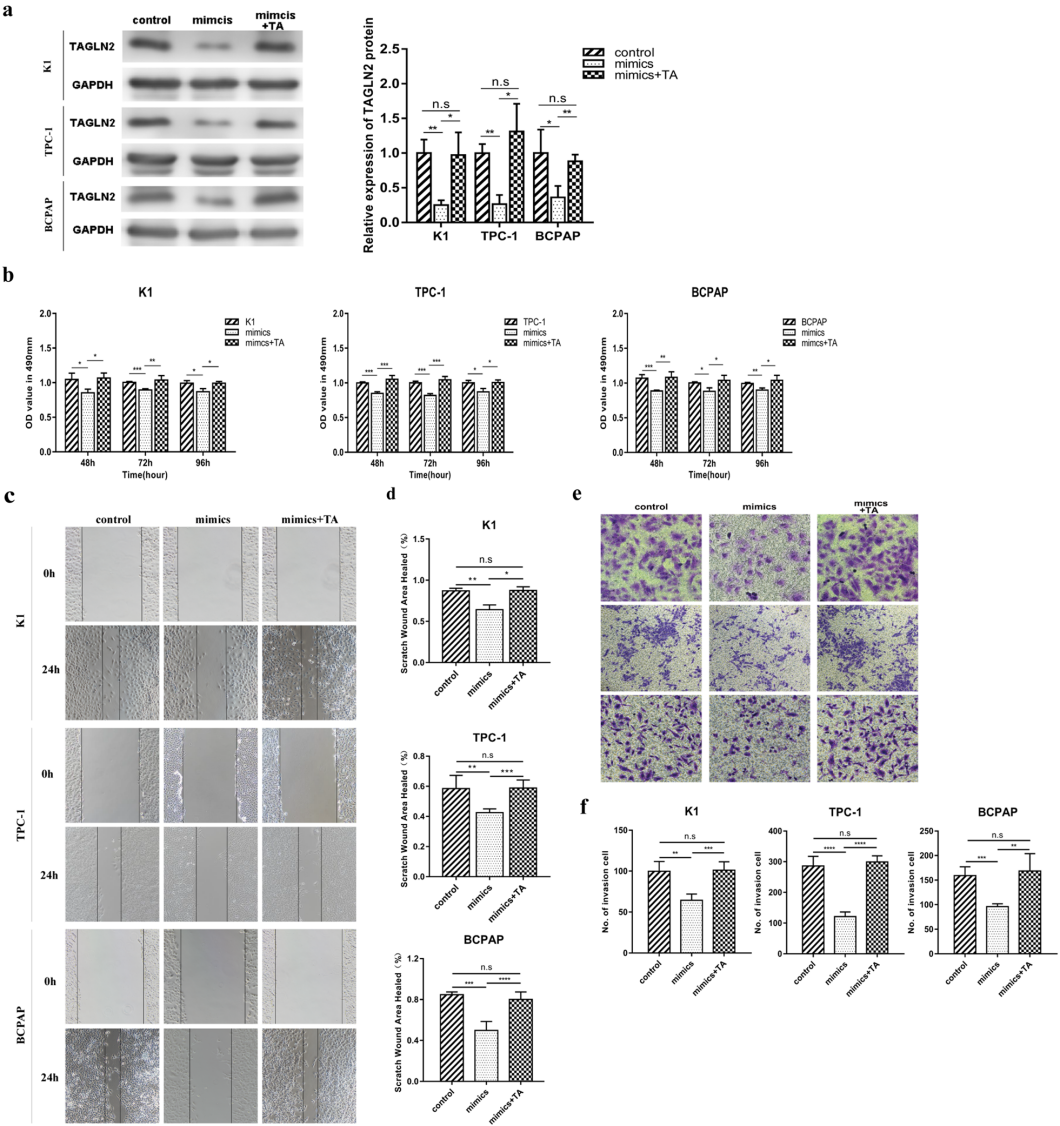
Inhibition of papillary thyroid cancer (PTC) cell proliferation, migration, and invasion by miR-613 directly targeting TAGLN2. A rescue experiment was performed to confirm that miR-613 exerts its inhibitory effect by targeting TAGLN2. **a** Significant re-upregulation of TAGLN2 in PTC cell lines after TAGLN2 overexpression based on western blot analysis. **b** Reversal of the inhibitory effects of miR-613 on PTC cell growth at 48, 72, and 96 h by TAGLN2 based on MTS assay. **c**, **d** Reversal of the inhibitory effects of miR-613 on cell migration by TAGLN2 based on wound healing assays. Representative images showing the migration of three PTC cells in six-well culture plates (**c**). Quantification of the cell migration distances corresponding to three PTC cells (**d**). **e**, **f** Reversal of the cell invasion inhibitory effects of miR-613 by TAGLN2. Images of cells that migrated to the bottom chamber (**e**), and calculated number of invasion cells (**f**). * p < 0.05, **p < 0.01, *** p < 0.001, **** p < 0.0001

Reportedly, TAGLN2 plays an important role in malignant tumor invasion and metastasis [35, 36, 38, 42–44]. In this study, to determine whether miR-613 participates in PTC cell invasion and metastasis by regulating the expression of *TAGLN2*, PTC cells were co-transfected with pCMV-TAGLN2 and miR-613 mimics. PTC cells transfected with miR-613 only and blank PTC cells were used as controls. As shown in Fig. 5a, TAGLN2 was significantly downregulated in PTC cell lines after transfection with miR-613 mimics, and its expression was re-upregulated after co-transfection with miR-613 mimics and pCMV-TAGLN2. Further, as indicated in Fig. 5b and Additional file 2: S2, the use of MTS assay to evaluate the proliferation ability of PTC cells showed a significant difference in proliferation between the group transfected with pCMV-TAGLN2 and miR-613 mimics and the group transfected with miR-613 mimics only. Furthermore, the group transfected with pCMV-TAGLN2 and miR-613 mimics and the group transfected with miR-613 mimics only showed significant differences in migration and invasion, suggesting that possibly, the upregulation of TAGLN2 partially offset the effects of miR-613 (Fig. 5c–f). The results of the migration and invasion assays are summarized in Table 3. Overall, the regulatory effects of miR-613 on the progression of PTC may be mediated by TAGLN2.

**Table 3:** The healing area and numbers of invasion cells in group of PTC cells transfected with mimics or mimics + TA

Cell lines	Scratch wound area healed	P value	Number of invasion cells	P value
K1				
Control	87.1% ± 3.0%	0.008^a^	99.8 ± 5.9	0.002^a^
Mimics	64.2% ± 5.7%	0.012^b^	64.3 ± 3.8	0.001^b^
Mimics + TA	87.6% ± 4.4%	0.930^c^	101.0 ± 5.2	0.880^c^
TPC-1				
Control	58.5% ± 3.9%	0.004^a^	286.0 ± 15.5	0.000^a^
Mimics	42.5% ± 1.1%	0.000^b^	121.0 ± 7.5	0.000^b^
Mimics + TA	58.9% ± 2.3%	0.925^c^	299.3 ± 10.0	0.500^c^
BCPAP				
Control	84.8% ± 1.2%	0.000^a^	159.3 ± 8.9	0.001^a^
Mimics	49.8% ± 3.9%	0.001^b^	96.0 ± 2.9	0.007^b^
Mimics + TA	80.1% ± 3.6%	0.220^c^	168.5 ± 17.7	0.657^c^

^a^Control vs. mimics

^b^Mimics vs. mimics + TA

^c^Control vs. mimics + TA

### Rescue of miR-613-induced EMT inhibition in PTC cells by TAGLN2

**Fig. 6:**
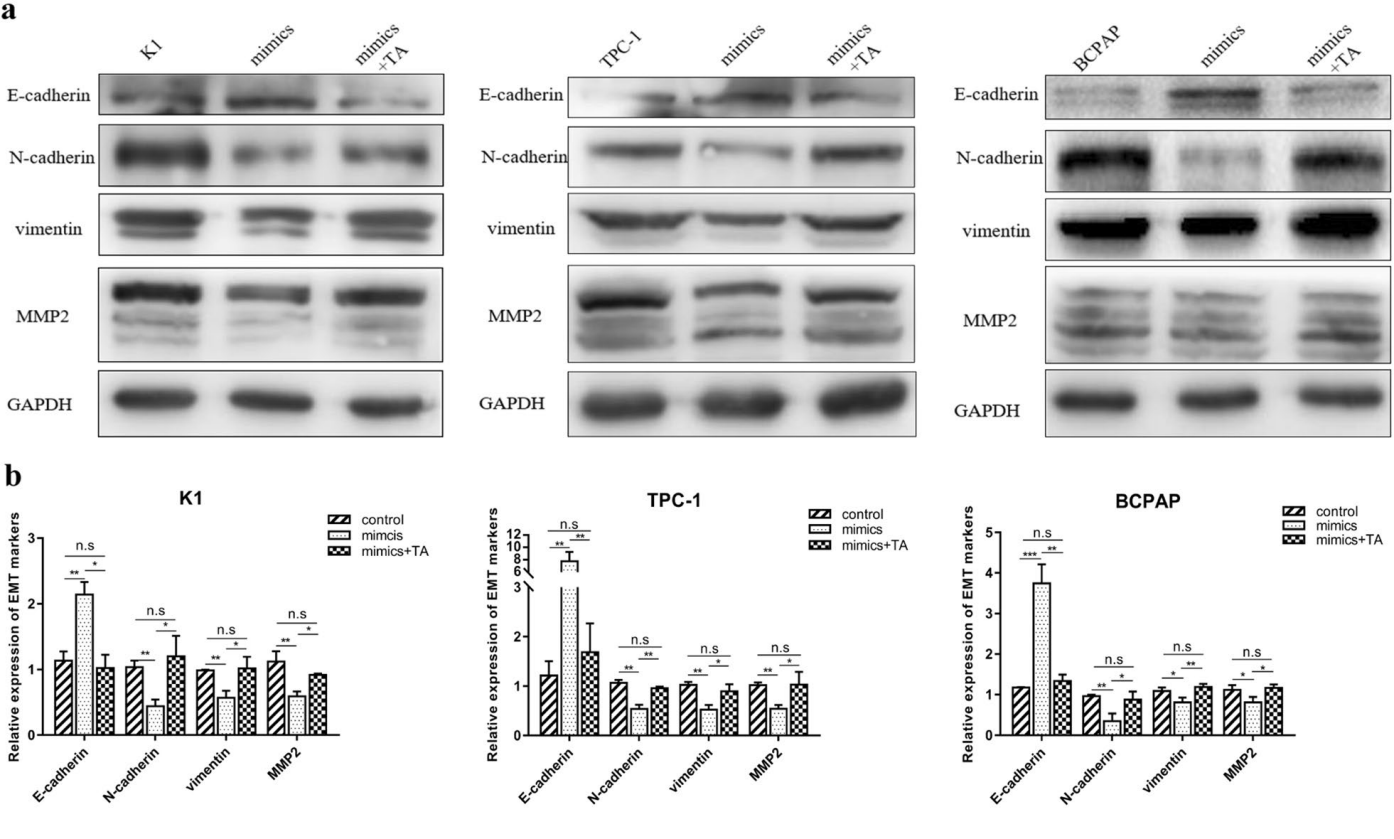
Inhibition of EMT by miR-613 via the direct targeting of TAGLN2 in papillary thyroid cancer (PTC) cells. K1, TPC-1, and BCPAP cells were transfected with miR-613 mimics or mimics + TAGLN2.** a** Decreased E-cadherin expression level and increased N-cadherin, Vimentin, and MMP2 expression levels in mimics group compared with the mimics + TAGLN2 group. **b** Relative expression levels of EMT-related markers. * p < 0.05, **p < 0.01, *** p < 0.001, **** p < 0.0001

As previously suggested, miR-613 regulates the expression of *TAGLN2* by binding to its 3′-UTR segment. We further evaluated whether TAGLN2 is able to rescue the inhibitory effect of miR-613 on EMT in PTC cells. Thus, it was observed that the protein expression levels of E-cadherin were significantly lower, while those of N-cadherin, vimentin, and MMP2 were significantly higher in PTC cells co-transfected with pCMV-TAGLN2 and miR-613 mimics than in cells transfected with miR-613 only. These findings indicated that the upregulation of TAGLN2 in PTC cells partially rescued the miR-613-induced inhibition of EMT (Fig. 6a and b). Collectively, these results demonstrated that miR-613 inhibits EMT by suppressing the expression of TAGLN2.

## Discussion

PTC is one of the most common endocrine malignancies, and over the last 25 years, its incidence has tripled [1, 2]. It is also associated with a high risk of early lymph node metastasis and the absence of specific symptoms. These factors make its diagnosis and treatment difficult. Further, the risk of metastasis occurring in the early stage of PTC increases the risks of recurrence and mortality [45–47]. Therefore, effective therapy and early diagnosis are crucial.

MiRNAs, which function as oncogenes or antioncogenes, and are candidate therapeutic and diagnostic targets for malignant tumors, have been a major focus of recent research efforts [48, 49]. The potential therapeutic anti-tumor effect of miRNAs has been confirmed in an increasing number of studies, and a series of clinical trials has been conducted in this regard. These studies have shown that the dysregulation of miRNA can be reversed by agomirs or antagomirs [50–52]. Recently, it was reported that miR-613 dysregulation, which is associated with cancer cell aggressiveness and progression, occurs in several types of malignant cancers [53, 54]. Further, it has been observed that the overexpression of miR-613 in hepatocellular cancer cells inhibits cancer stem cell expansion and increases sensitivity to cisplatin and sorafenib [30]. Particularly, in gastric cancer, miR-613 overexpression suppresses GC cell proliferation, cell cycle progression, and migration [29]. Additionally, in triple-negative breast cancer cells, miR-613 possibly inhibits cell migration and invasion [28]. However, its function in PTC is still unclear. The results of this study demonstrated that miR-613 levels were markedly downregulated in patients with PTC and were significantly lower in PTC with lymph node metastasis than in tissues without lymph node metastasis. These results suggest that low miR-613 expression levels may be associated with PTC invasion and metastasis.

Further, we observed that miR-613 was markedly downregulated in PTC cell lines, and its upregulation resulted in a significant inhibition of PTC cell proliferation, migration, and invasion. EMT is known to play an important role in cell morphology and motility, and during such processes, epithelial cells lose their adhesion properties and acquire mesenchymal features alongside the dysregulation of a series of phenotypic biomarkers, under the action of various inducers. Consequently, the rate of cell metastasis increases, and metastasis to distant tissues and organs can also occur [55]. To determine the mechanism by which miR-613 inhibited PTC cell invasion, we evaluated the expression levels of crucial EMT-related proteins (E-cadherin, N-cadherin, vimentin, and MMP2) after the overexpression of miR-613. E-cadherin was observed to be significantly upregulated, while N-cadherin, vimentin, and MMP2 were markedly downregulated, indicating that miR-613 may be a negative regulator of EMT in PTC cells. These results suggested that the restoration of miR-613 expression may be an effective strategy by which PTC progression can be controlled.

As important gene expression regulators, miRNAs participate in physiological or pathological processes by regulating protein expression at the post-transcriptional level. Therefore, the identification of miRNA targets is crucial for the elucidation of cancer pathogenesis. Using various prediction algorithms, *TAGLN2* was identified as a direct target of miR-613. Specifically, we observed that the expression of TAGLN2 was upregulated in PTC tissues and was negatively related to the expression of miR-613. Further, in PTC cell lines, the overexpression of miR-613 resulted in a decrease in the levels of TAGLN2. A dual-luciferase reporter assay also confirmed that TAGLN2 is a direct target of miR-613.

TAGLN2, an actin-binding protein, contributes to the formation of the cytoskeletal structure and helps in the maintenance of its stability; this is fundamental for a wide range of biological processes [56]. TAGLN2 is also overexpressed in various cancers, and its dysregulation is associated with the progression of malignant tumors [37, 39, 57–59]. In this study, rescue experiments revealed that TAGLN2 overexpression attenuates the inhibitory effects of miR-613 on PTC cell proliferation, migration, and invasion. According to a previous study, TAGLN2 silencing specifically inhibits F-actin-rich compartments in glioma cells, thereby reducing the formation of pseudopodia during cell invasion [37]. In non-small-cell lung cancer, hypoxia-inducible TAGLN2 can promote EMT by activating the IGF1RB/PI3K/AKT pathway, thereby stabilizing Snail1, which functions as an E-cadherin suppressor [36]. These previous findings indicate that TAGLN2 may be a crucial regulator of metastasis and aggressiveness in malignancies. To further clarify the role of TAGLN2 in the invasive pathological process of PTC, PTC cells were co-transfected with miR-613 mimics and TAGLN2, and analyses were performed. Thus, it was observed that the miR-613-induced suppression of EMT was restored by the overexpression of TAGLN2. These results demonstrate the vital effects of miR-613-driven TAGLN2 suppression on the inhibition of PTC progression. Furthermore, the results of this study showed that miR-613 regulated EMT-related proteins by regulating TAGLN2 expression, suggesting that TAGLN2 may act as a bridge between upstream regulators and downstream effectors. Thus, the effect of TAGLN2 on morphological changes and the underlying mechanism related to EMT remain to be determined. In future, we will explore the molecular pathways that are associated with the involvement of TAGLN2 in morphological changes and also reveal the precise molecular mechanism underlying PTC invasion and metastasis.

## Conclusion

In this study, we obtained evidence that miR-613 inhibits PTC cell proliferation, metastasis, and EMT by downregulating TAGLN2 expression. Overall, our findings indicated that miR-613 is a potential clinical biomarker for PTC progression and could serve as a therapeutic target for human PTC. In future, we plan to conduct an in-depth investigation into the role of TAGLN2 in PTC and clarify the molecular mechanisms by which TAGLN2 regulates the progression of PTC.

## Supplementary Information

**Additional file 1: Figure S1.** The representative image color changes at each time point. As shown in Figure S1, the color depths corresponding to the mimics group at 48, 72 and 96 h were lighter than those corresponding to the control and NC groups at the same time points.

**Additional file 2: Figure S2.** The representative image color changes at each time point. As shown in Figure S2, the color depths corresponding to the mimics group at 48, 72 and 96 h were lighter than those corresponding to the control and mimics + TA groups at the same time points.

## Data Availability

Not applicable.

## Abbreviations

PTC: Papillary thyroid cancer
EMT: Epithelial–mesenchymal transition
miRNA: MicroRNA
3′-UTR: 3′ Untranslated region
PSMD10: Proteasome 26S subunit non-ATPase 10
ARFGEF1: ARF Guanylate exchange factor
AKT: Protein kinase B
GSK-3β: Glycogen synthase kinase 3β
MMP2: Matrix metalloproteinase 2
PI3K: Phosphatidylinositol-4, 5-bisphosphate 3-kinase
IGF1RB: Insulin-Like Growth Factor I Receptor, beta
