# Supplementary figures and images for: MiR-613 inhibits the proliferation, migration, and invasion of papillary thyroid carcinoma cells by directly targeting TAGLN2
Source: Cancer Cell Int. 2021 Sep 16;21:494. doi: 10.1186/s12935-021-02083-8 (PMC8447791; doi:10.1186/s12935-021-02083-8)

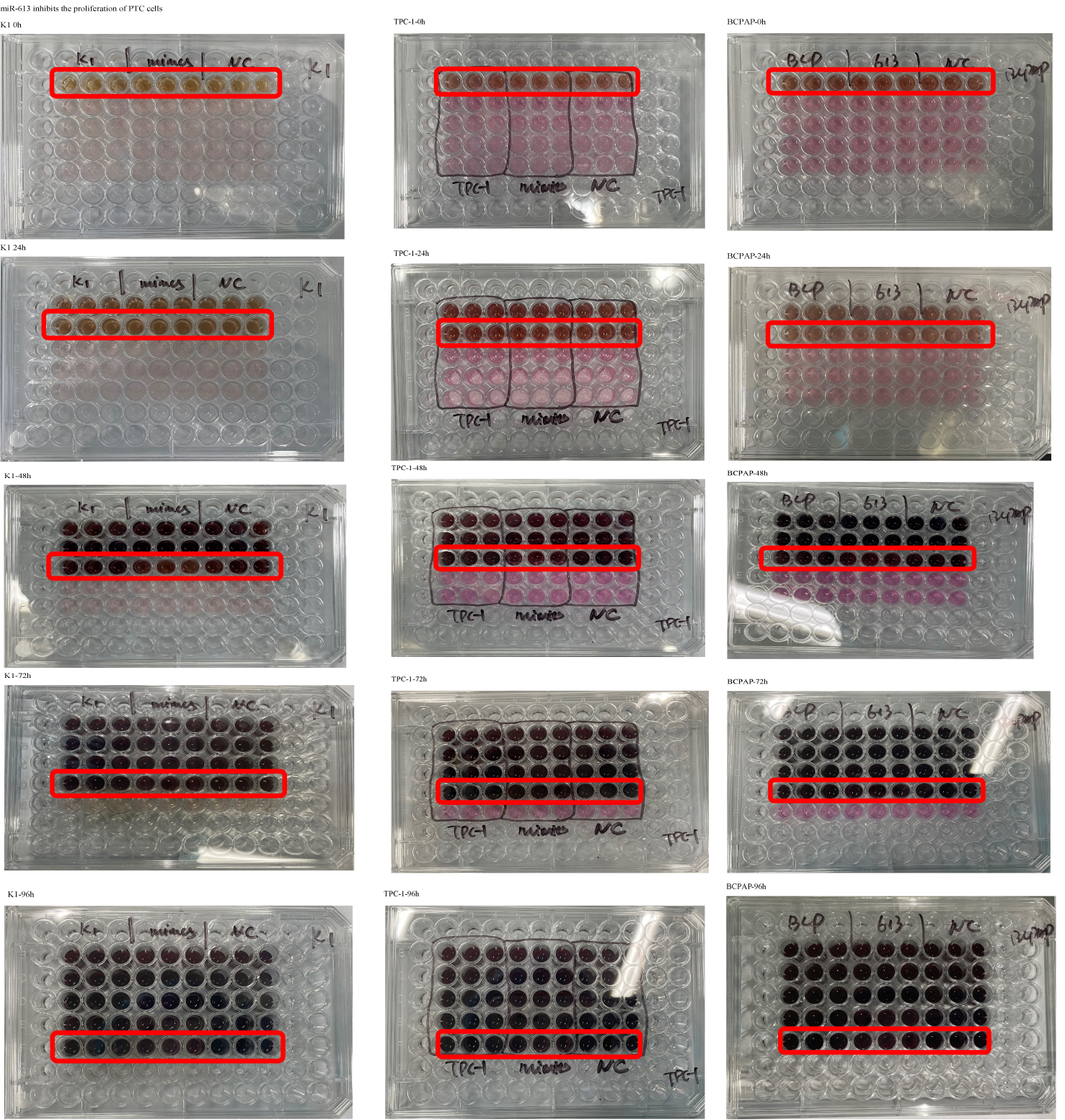

Supplement: Supplementary file 1 — Additional file 1: Figure S1. The representative image color changes at each time point. As shown in Figure S1, the color depths corresponding to the mimics group at 48, 72 and 96 h were lighter than those corresponding to the control and NC groups at the same time points. [file 12935_2021_2083_MOESM1_ESM.tif]

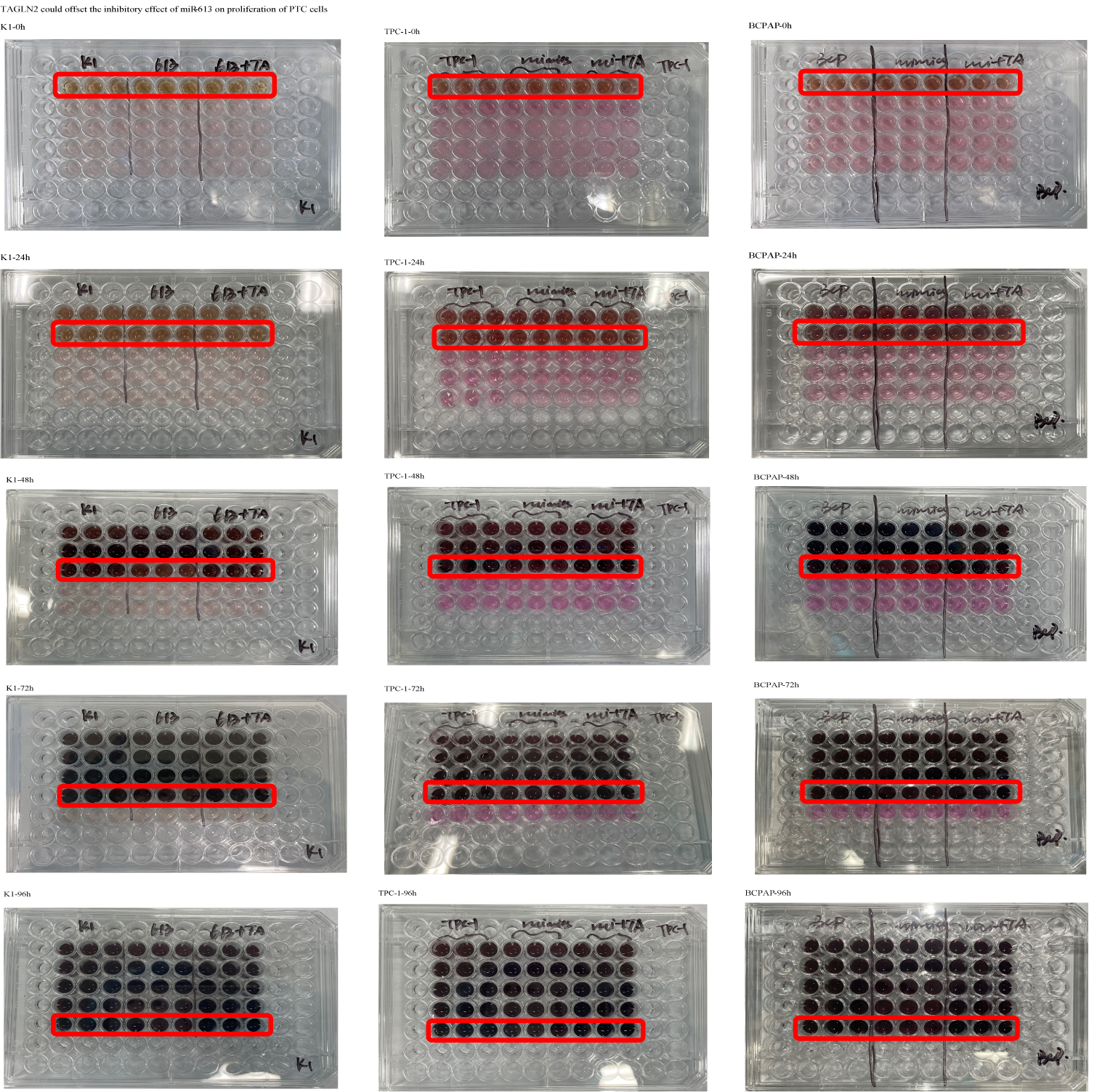

Supplement: Supplementary file 2 — Additional file 2: Figure S2. The representative image color changes at each time point. As shown in Figure S2, the color depths corresponding to the mimics group at 48, 72 and 96 h were lighter than those corresponding to the control and mimics + TA groups at the same time points. [file 12935_2021_2083_MOESM2_ESM.tif]
